# Neuroprotective Effect of Solid Lipid Nanoparticles Loaded with *Lepidium sativum* (L.) Seed Bioactive Components Enhance Bioavailability and Wnt/β-Catenin/Camk-II Signaling Cascade in SH-SY5Y Neuroblastoma Cells

**DOI:** 10.3390/nano14020199

**Published:** 2024-01-16

**Authors:** Nada Al-Saran, Pandurangan Subash-Babu, Laila Naif Al-Harbi, Bahauddeen M. Alrfaei, Ali A. Alshatwi

**Affiliations:** 1 Department of Food Science and Nutrition, College of Food and Agricultural Sciences, King Saud University, P.O. Box 2460, Riyadh 11451, Saudi Arabiasubash@ksu.edu.sa (P.S.-B.);; 2 College of Medicine, King Saud Bin Abdulaziz University for Health Sciences (KSAU-HS), Minister of National Guard-Health Affairs (MNGHA), P.O. Box 22490, Riyadh 11426, Saudi Arabia; 3 King Abdullah International Medical Research Center, Minister of National Guard-Health Affairs (MNGHA), P.O. Box 22490, Riyadh 11426, Saudi Arabia

**Keywords:** SLNP, A*β* (1-42) fibrils, oxidative stress, A*β*-neurotoxicity, Wnt/Camk-II, post synapse

## Abstract

The primary pathological hallmark of Alzheimer’s disease (AD) is the formation and accumulation of neurofibrillary tangles and plaques, which result from the aggregation of amyloid-*β* (A*β*) induced by oxidative stress. The effectiveness of Alzheimer’s disease (AD) therapeutics significantly hinges on the drug’s bioavailability and its ability to penetrate neuronal cells. The current investigation was designed as a first attempt to examine bio-fabricated *Lepidium sativum* (LS) seed-extract-loaded solid lipid nanoparticles (SLNps) to increase bioavailability and bioefficacy for the prevention of undifferentiated SH-SY5Y neuronal cells from oxidative stress induced by H_2_O_2_ and amyloid-*β* peptide (A*β*,1-42). The SLNps were fabricated using LS extract as a water phase and hyaluronic acid and chia seed fatty acids as a lipid phase, then confirmed and characterized using UV, Zeta size, and SEM methods. The biological safety of synthesized LS-SLNps has been determined using MTT assay and PI staining (nuclear damage) in hMSCs. LS-SLNp-pretreated neuronal cells were induced with oxidative stress and 2 µM of beta-amyloid (A*β*,1-42) fibrils; furthermore, the neuroprotective potential of LS-SLNps was determined through the quenching of oxidative stress, enhancing mitochondrial oxidative capacity, and immunoregulatory potential. Observations found that cells treated with both H_2_O_2_ and beta-amyloid (A*β*,1-42) fibrils showed decreased neuronal cell growth, nuclear damage, and mitochondrial membrane potential due to oxidative stress. However, SH-SY5Y cells pretreated with LS-SLNps for 24 h showed an increase in cell proliferation with uniform morphology and increased mitochondrial membrane potential compared to cells pretreated with LS alone. Gene expression analysis found that LS-SLNps increased the expression of Wnt 3a and 5a, which stimulated the canonical, β-catenin, and non-canonical Camk-II expressions of nerve cell growth factors, confirming the molecular-level reversal of neurodegenerative diseases.

## 1. Introduction

Neuronal cell death mainly occurs after oxidative stress, involving apoptosis induced by alterations in the brain cell’s internal microenvironment. This process is implicated in the onset of neurodegenerative conditions like Alzheimer’s disease (AD) or cerebral ischemia [1]. Molecular mechanisms related to pathological neurodegenerative disease share some standard features, such as the accumulation of misfolded proteins, oxidative damage to DNA, neuro-excitotoxicity, and neuroinflammatory responses [2]. Amyloid-*β* (A*β*,1-42) is the aggregated misfolded protein considered to be the principal hallmark in many neurodegenerative diseases. AD patients have been observed to have a high deposition of A*β* (1-42) fibrils in their brain, a depressed Wnt signaling cascade, and intracellular hyperphosphorylated Tau (p-Tau) [3].

During cellular respiration, the release of free electrons from the electron transport chain (ETC) is unavoidable. These free electrons bind to oxygen to generate superoxide anions (O^2−^), collectively referred to as reactive oxygen species (ROS) [4]. In particular, the energy uptake and survival of nerve cells majorly depend on aerobic oxidative respiration [5]. Consequently, the rich lipid content, high energy demand, and availability of low-molecular-weight antioxidants in the brain are associated with excessive oxidative insults [6]. In the brain, oxidative stress (OS)-induced damage is more pronounced in comparison to other organs. This high susceptibility is attributed to the brain’s excessive oxygen demand, the presence of abundant redox-active iron and copper metals, and the abundant oxidation of mitochondrial aerobic polyunsaturated fatty acids [7,8]. Prolonged oxidative insult is implicated in neuronal cell apoptosis, contributing to the onset of neurodegenerative diseases such as AD, Parkinson’s disease, and cerebral ischemia [5].

Naturally occurring dietary polyphenols can exert antioxidant and anti-inflammatory benefits. Red wine, grape seeds, and pomegranate-derived polyphenols, such as resveratrol and ellagitannins, have been identified for their neuroprotection in both in vitro and in vivo studies [9,10]. However, the direct impact of these polyphenols on neuronal cells is a subject of debate due to their limited bioavailability. Polyphenols face challenges in terms of poor bioavailability, hindering their ability to reach systemic tissues [11,12]. Since the majority of phenolics undergo metabolism and do not reach the nervous system or brain in the same form as they occur in dietary sources, their direct impact is limited. Alternatively, gold nanoparticles (AuNPs) have been reported as providing benefits for human neural stem cells (hMSCs) treated with *β*-amyloid (A*β*). However, the specific neuroprotective mechanisms remain unclear. In this approach, Shivananjegowda et al. [13] found that SLNps formulated using memantine hydrochloride (MeHCl) and tramiprosate (TMPS) confirmed the metabolic degradation of A*β* on SH-SY5Y cells.

*Lepidium sativum* L. is a commonly used medicinal plant consumed as a health improver, and it is used as an antioxidant to recover from illness [14]. Previously, alkaloids present in the seeds and aerial parts of *Lepidium sativum* L. (LS) have been identified as having antioxidant, hepatoprotective, and anti-inflammatory potential [15,16]. The present study is a first attempt to fabricate polyphenol- and alkaloid-rich LS seed-extract-loaded SLNps (LS-SLNps) to increase bioavailability and analyze the bioefficacy in the prevention of SH-SY5Y neuronal cells from amyloid-*β* peptide (A*β*,1-42) and H_2_O_2_-induced oxidative stress. The influence of SLNps on quenching of oxidative stress, enhancing mitochondrial oxidative capacity, clearance of A*β* (1-42) fibril deposition, and stimulation of the molecular-level Wnt signaling pathway for neuronal cell growth have been analyzed.

## 2. Materials and Methods

### 2.1. Chemicals

MTT [3-(4,5-dimethyl-2-thiazolyl)-2,5-diphenyl-2H-tetrazolium bromide), phosphatidylcholine, hyaluronic acid, sialic acid, trypan blue, dimethyl sulfoxide (DMSO), amyloid-*β* peptide (A*β*,1-42), and hydrogen peroxide (H_2_O_2_) were obtained from Sigma-Aldrich (St. Louis, MO, USA). Propidium iodide, phosphate-buffered saline (PBS), jc-1 (MMP assay), acridine orange, ethidium bromide, and molecular biology-related chemicals were acquired from Sigma-Aldrich (St. Louis, MO, USA).

### 2.2. Plant Material Collection, Extraction of Lepidium sativum L. (Cress Seed) and Salvia hispanica. L. (Chia Seed)

*Lepidium sativum* L. (cress seed) and *Salvia hispanica* L. (chia seed) were purchased from iHerb (Moreno Valley, CA, USA). In accordance with national and international regulations, the plant species employed in the present study were identified and authenticated by a taxonomist. After the identification process, the voucher specimens for *Lepidium sativum* L. (KSU-LS-15) and *Salvia hispanica* L. (KSU-SH-07) were preserved in the Public Herbarium, College of Science, King Saud University, Riyadh-11451.

*Lepidium sativum* L. (cress seed) and *Salvia hispanica* L. (chia seed) were ground for 30 s, respectively. In a glass container, 400 gm of *Lepidium sativum* L. (LS) powder was weighed, 1.2 L of methanol was added to the container, and kept for 72 h in a mechanical shaker at 30 rpm. After 72 h, the solute was separated using Whatman No. 1 filter paper and the solvent was separated using a rotary evaporator under reduced pressure at 50 °C. Condensed LS extract was collected and preserved in a brown container at −20 °C until further use. Separately, 200 gm of coarsely powdered *Salvia hispanica* L. seed (SH) was immersed in 600 mL of ethyl acetate for 72 h allowed for shaking using a fixed shaker. The extraction progress of *Salvia hispanica* L. seeds has been carried out in the same way as the LS seed progress.

### 2.3. Phytochemical Identification

GC-MS (Agilent 7890A; Agilent Technologies, Santa Clara, CA, USA) was used to analyze the extract(s) and identify their chemical compounds. The concentration of each compound was expressed as a peak area percentage. A DB-5MS (30 m\0.250 mm\0.25 µm) column was used. The flow rate was fixed at 1 mL/min, the pressure was set at 10.42 psi, splitless mode, and the injection volume was 3 µL. The oven temperature was initiated with 60 °C for 3 min, 100 °C for 1 min (a rate of 3 °C/min), 200 °C for 1 min (a rate of 3 °C/min), and 300 °C for 1 min (a rate of 5 °C/min). NIST libraries were used to interpret GC-MS data. The chromatogram threshold was set at 17 throughout all experiments.

### 2.4. Preparation of L. sativum Seed Extract Containing Solid Lipid Nanoparticle (LS-SLNp)

SLNp were prepared according to the emulsification evaporation principle, LS-loaded with hyaluronic acid (HA), *Salvia hispanica* seed phospholipid (SHE, having omega 3: omega 6 as a 3:1 ratios), phosphatidylethanolamine, palmitic acid, and stearic acid, as described by Xue et al. [17], with a minor modification. Briefly, the lipid phase containing 10 mg of palmitic acid and 10 mg of stearic acid was dissolved in 20 mL of acetone, hyaluronic acid, phosphatidylethanolamine (10 µL), and solvent-free SHE-phospholipids (15 µL), LS (25 mg) were dissolved entirely using a magnetic stirrer at 70 °C. The preheated aqueous phase containing Tween 20 has emulsified with the warm organic lipid phase (30 mg dissolved in 15 mL of water). The whole procedure was carried out at 70 °C (melting temperature for lipid) using a hot plate magnetic stirrer with continuous stirring for 60 min. The oil-in-water dispersion was sonicated using a probe-type Ultrasonicator (Sonics, Newtown, CT, USA) in an ice bath at a frequency of 0.5 cycles with 60% amplitude. The formulation contains 20 mL of 30 mg lipid phase, 15 mL of the aqueous phase, and 0.05% of the LS extract. The obtained dispersion was collected and stored in a brown glass container (2–4 °C).

### 2.5. Characterization of Prepared LS-SLNp

The concentration of loaded drug in LS-SLNp was determined by disrupting 1 mL of freshly prepared LS-SLNp and absorbance were measured at 420 nm using a spectrophotometer. Chemical interactions, functional groups with a carbon–oxygen double bonds, and appearance of chemical (aldehyde and ketone) groups with other excipients were documented through Fourier-transform infrared spectroscopy (FT-IR) equipment from Agilent, Santa Clara, CA, USA. The z-average diameter for particle size was determined using the dynamic light scattering (DLS) technique with a Zetasizer (NANO-Zs90). Morphological analysis involved placing 5 μL of LS-SLNp on a 300-mesh carbon-coated copper grid, with negative staining achieved using 2% uranyl acetate (*w*/*v*). High-resolution scanning electron microscopy (SEM) from JEOL, Japan, was utilized to examine the morphology (shape and size) from the stained grids.

### 2.6. Cell lines and Cell Culture

Human neuroblastoma (SH-SY5Y) cell and human mesenchymal stem cell (hMSCs) lines were obtained from the American type culture collections (ATCC, Manassas, VA, USA). SH-SY5Y cells were cultured using growth media (Thermo Fisher Scientific, Waltham, MA, USA) containing, 10% FBS (fetal bovine serum), DMEM (Dulbecco’s Modified Eagle’s Medium), 2 mM _L_-glutamate and antibiotic (1% penicillin/streptomycin) combination in 5% CO_2_ at 37 °C conditions. Once 80% confluence was visually confirmed, the media was gently removed, and the cells were washed with Dulbecco’s phosphate-buffered saline (DPBS) solution. Subsequently, cells were harvested after trypsinization using 0.25% trypsin/EDTA solution. Further, the cell’s pellet was collected after 5 min centrifugation at 3500 rpm at room temperature and counted using a hemocytometer. The respective number of cells were seeded to 96 well (1 × 10^4^ cells/well) plate for MTT assay; or 24 well (5 × 10^4^ cells/well) plate for cellular staining, and cDNA synthesis for gene expression analysis.

### 2.7. Preparation of Beta-Amyloid (Aβ,1-42) Peptides

Beta-amyloid (A*β*,1-42) peptides were dissolved in 1 mM of hexafluoro isopropanol and the aliquots stored in a sterile brown glass container. Then, hexafluoro isopropanol was removed under vacuum pressure; furthermore, the pure A*β* (1-42) peptide fibrils were collected and stored at −20 °C. The peptides were first suspended in dry DMSO to a concentration of 5 mM. Working concentration of A*β* (1-42) was prepared using 10 mM HCL to obtain 100 µM of peptide fibrils and incubated for 24 h at 37 °C.

### 2.8. Biocompatibility Assessment of LS and LS-SLNp in hMSCs and SH-SY5Y Cells

To assess the biosafety of LS and LS-SLNp, the hMSCs and SH-SY5Y cells were exposed with 0, 5, 10, 20, 40, and 80 µg/100 mL concentrations of LS and LS-SLNp for 48 h, respectively. Following incubation, 20 µL/well of MTT (5 mg/mL) was added and maintained for 4 h at 37 °C in a CO_2_ incubator. After incubation, the purple formazan crystals were confirmed and dissolved using 100 µL of DMSO (100%). The quantity of formazan compound was measured using a microplate reader (Thermo Scientific, Waltham, MA, USA) with the absorbance at 570 nm. The quantity of viable cells were evaluated in percentage according to the average value of absorbance of the sample/absorbance of the control × 100.

### 2.9. Assessing the Toxic Dose for H_2_O_2_ and Aβ (1-42) Using MTT Assay

SH-SY5Y cells (1 × 10^4^ cells/well) were allowed to grown in 96 well plates. To determine the inhibitory dose of H_2_O_2_ and A*β* (1-42), increasing concentrations of H_2_O_2_ (0, 0.6, 1.2, 2.5, 5, 10, 20, and 40 mM) and A*β*,1-42 (0, 0.25, 0.5, 1, 2, 4, 8, and 16 µM) were treated to the cells (after 80% confluence) for 24 h, respectively. After incubation, according to the MTT principle and methodology, the percentage of viable cells was calculated.

### 2.10. Neuroprotective Effect of LS-SLNp in H_2_O_2_ and Aβ (1-42) Induced SH-SY5Y Cells

Further, SH-SY5Y cells were pretreated with increasing concentrations (0, 0.5, 1, 2, 4, 8, and 16 µg/100 mL) of LS and LS-SLNp for 24 h, respectively. The pretreated cells were exposed with the toxic doses of H_2_O_2_ (10 mM) and A*β*,1-42 (2 µM) in all wells and incubated for next 24 h to identify the neuroprotective effect; untreated cells were considered as negative controls; and SLNp-alone-treated cells were considered as positive control. After incubation, according to the MTT principle and methodology, the percentage of viable cells was calculated.

## 3. Experimental Design

**Figure 1:**
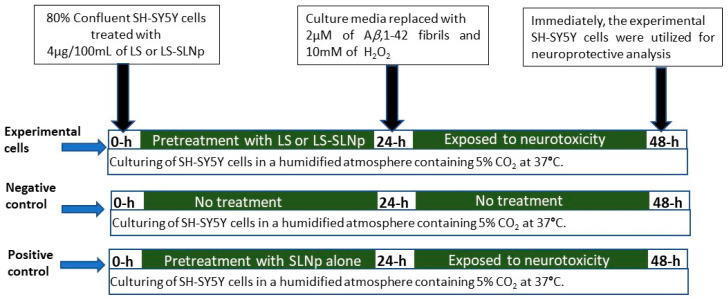
Experimental design of the present study.

The experiment was intended to analyze the protective effect of 4 µg/100 mL of LS or LS-SLNp against SH-SY5Y cells exposed to neurotoxic agents (using H_2_O_2_ oxidative stress and A*β*,1-42 fibrils) through analyzing the cellular and nuclear characteristics, pro-inflammation, and apoptosis-related gene expression (Figure 1).

To determine the neuroprotective potential, SH-SY5Y cells were pretreated with 4 µg/100 mL of LS and LS-SLNp for 24 h, respectively. Further, pretreated cells were incubated with 2 µM of beta-amyloid (A*β*,1-42) fibrils and 10 mM of H_2_O_2_ for the following 24 h. Negative control (SH-SY5Y cells cultured in growth media alone added with 0.01% DMSO as solvent control) and positive control (SH-SY5Y cells exposed to oxidative stress and beta-amyloid toxicity with SLNp alone) have been maintained separately. After 48 h, the negative control, positive control and experimental cells were examined using an inverted microscope for nuclear damage using a light or fluorescence microscope. Another set of positive control and experimental cells have been used for protein quantification and gene expression analysis.

### 3.1. Cell and Nuclear Morphology

SH-SY5Y cells were cultured at a density of 5 × 10^4^ cells/well in a 24 well plate for this experiment. According to the experimental protocol, positive control, LS, or LS-SLNp (4 µg/100 mL) pretreated SH-SY5Y cells were exposed to neurotoxic agents (10 mM of H_2_O_2_ and 2 µM of beta-amyloid (A*β*,1-42) fibrils). Following 48 h incubation, the treated cells underwent processing to assess apoptotic and necrotic morphological changes. This analysis was conducted using either light microscopy or florescent microscopy (with propidium iodide (PI), acridine orange (AO) and ethidium bromide (EO)), following the methods outlined by Leite et al. [18]. Briefly, the culture media were gently removed without disturbing the adherent cells, followed by a gentle wash with 1% PBS. For nuclear morphology, 1 mg/mL of propidium iodide (PI; Sigma, St. Louis, MO, USA) solution was applied, then kept in the dark for 20 min at 37 °C. After the incubation period, unbound dye was gently washed away with PBS, and the stained cells were promptly examined under an inverted fluorescent microscope (Carl Zeiss, Jena, Germany) equipped with a green filter. Images were captured at a magnification of 200×.

In addition, to determine the apoptosis and necrosis the dual fluorescent staining solution (1:1 ratio) containing AO (100 μg/mL) and EB (100 μg/mL) (AO/EB; Sigma, St. Louis, MO, USA) solution was applied until the cells were completely immersed and then covered with a coverslip. Within 20 min, the morphology of early apoptotic, pre-apoptotic, late apoptotic, and necrotic cells were examined using a fluorescent microscope (Carl Zeiss, Jena, Germany). Subsequently, after the 20 min incubation period, the cells were gently rinsed with PBS to remove the dye, and the stained cells were promptly examined using fluorescent microscope. Images were captured at a magnification of 200×. The staining procedure was repeated a minimum of three times. The percentages of cells exhibiting apoptotic and necrotic morphology were manually calculated based on a random selection of 500 stained cells.

### 3.2. JC-1 Staining assay

The assessment of mitochondrial membrane potential (Δψ_m_) was conducted using the JC-1 staining assay. SH-SY5Y cells, cultured at a density of 5 × 10^4^ cells/well in 24 well plate, were subjected to pretreatments with a positive control, LS or LS-SLNp (4 µg/100 mL). In brief, JC-1 staining solution, mixed with an equal quantity of culture medium, was added to experimental cells. Further, the cells were incubated for 20 min in the dark at 37 °C. Following incubation, the unbound JC-1 dye was removed using 200 μL of wash buffer (specific for JC-1 staining) at 4 °C, and this process was repeated twice, then the JC-1 monomers (green, low MMP) and J-aggregate (red, high MMP) conversions were observed under fluorescence microscopy (200×). Randomly, 500 stained cells were characterized and Δψ_m_ was determined manually and images were captured.

### 3.3. Measurement of Pro-Oxidant and Antioxidant Levels in LS and LS-SLNp-Pretreated hMSCs and SH-SY5Y Cells Undergo Oxidative Stress

Pretreated hMSCs and SH-SY5Y cells with 4 µg/100 mL of LS or LS-SLNp present in 24 well plate, respective cells were exposed to oxidative stress using H_2_O_2_ for hMSCs, and 10 mM of H_2_O_2_ & 2 µM of A*β*,1-42 for SH-SY5Y for 24 h. The cells that underwent different treatment conditions were labelled accordingly; then, the cells were lysed in ice-cold lysis buffer (pH 7.4, 0.1 M Tris/HCL added with 0.5% Triton X-100, 5 mM β-mercaptoethanol, 0.1 mg/ mL serine protease inhibitor phenylmethylsulfonylfluoride). The cell lysate was collected in labelled microcentrifuge tubes and centrifuged at 14,000× *g* for 5 min at 4 °C. The supernatant was used to analyzed the levels of LPO, activities of glutathione reductase (GR), catalase (CAT), superoxide dismutase (SOD), and glutathione peroxidase (GPX) using an enzymatic commercial analytical kit (Sigma-Aldrich, St. Louis, MO, USA) and a multiwell plate reader. The assay protocol was followed according to the kit manual provided by the company (detailed methodology presented in the Supplementary Materials: Methodology—pro- and antioxidant assay).

### 3.4. Gene Expression Analysis

Total RNA and complementary DNA (Cdna) were synthesized from positive control, LS (4 µg/100 mL), or LS-SLNp (4 µg/100 mL) pretreated cells using Fastlane^®^ cell cDNA kit. Quantitative-PCR was performed using Applied Biosystems 7500 Fast Real-Time PCR System (Foster City, CA, USA). The mRNA expression levels of antioxidant, pro-inflammatory, and tumor suppressor in hMSCs, as well as neuronal inflammation and neuroprotective factors (Table 1) in SH-SY5Y cells, were quantified. The analysis including the reference gene, *β*-actin, was conducted following the method of Yuan et al. [19]. All the primers were purchased from Qiagen and their sequences were verified in primer-BLAST, National Library of Medicine, online software, and OriGene Global, Rockville, MD 20850, USA. Two negative controls were included for each gene by removing template cDNA. The relative expression levels were calculated by the formula: ΔΔCt (comparative threshold) = amplification values of LS-SLNp-treated (ΔCt) −ΔCt (positive control). Amplification of the target gene was normalized to its corresponding *β*-actin using the 2^−ΔΔCt^ method using Applied Biosystems with 21 CFR Part 11 software (version 2.0.4).

### 3.5. Quantification of Protein Using ELISA

The assessment of nerve cell inflammation and neuroprotective factors in SH-SY5Y cells, such as SFRP-1, T-tau, P-tau, TGF-β, β-catenin, and growth-associated protein (GAP-43), was conducted in positive control and LS-SLNp (4 µg/100 mL)-pretreated cells. This analysis was performed using high-sensitivity ELISA kits from Quantikine (R&D Systems, MN, USA). It is important to note that the assay does not differentiate between soluble and receptor-bound proteins, measuring the total concentration of proteins. The quantification of these proteins was expressed as pg/mg protein, providing a standardized measure across all the analyzed proteins.

### 3.6. Statistical Analysis

The experiments were carried out with 3 independent repetitions of each parameter. The data were acquired and presented a mean of 3 data points with SD (wherein each of the 3 data points was a mean of the 6 (n = 6) technical replicates). All data from the experimental groups underwent statistical evaluation using the SPSS/28.5 software package (IBM, New York, NY, USA). The analysis involved one-way analysis of variance (ANOVA) for the experimental groups. Subsequently, post-hoc analysis was performed using Tukey’s range test to compare and analyze the data within and between the groups. Statistical significance for all comparisons was set at *p* ≤ 0.05 and *p* ≤ 0.001, as indicated by the chosen level of significance [20].

## 4. Results

### 4.1. Characterization of LS-SLNp

**Figure 2:**
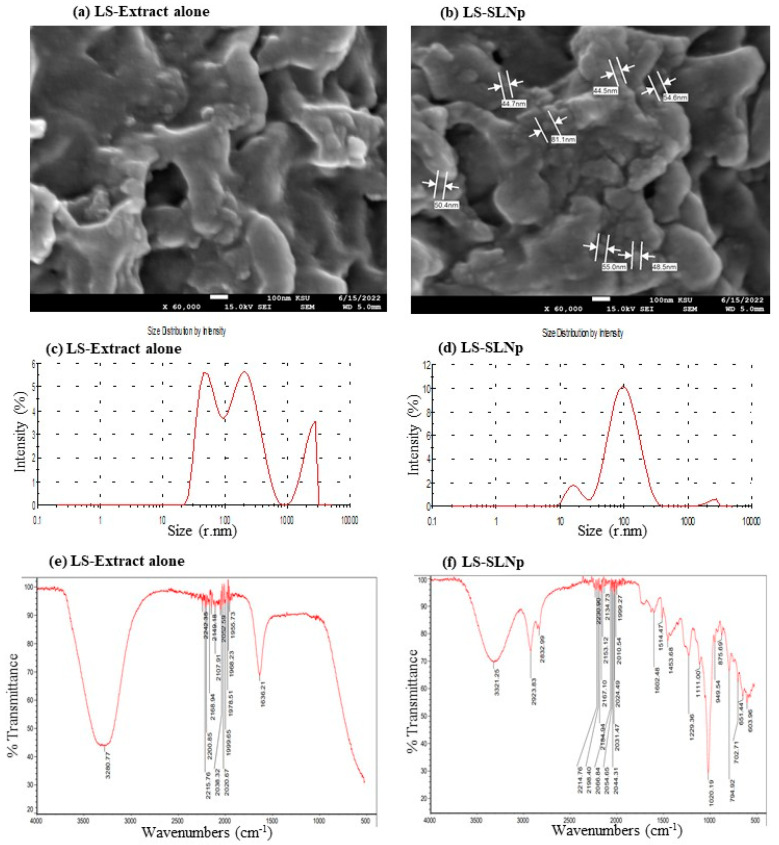
Characterization of *L. sativum* extract alone (**a**,**c**,**e**) and *L. sativum* extract-loaded solid lipid nanoparticle (LS-SLNp) (**b**,**d**,**f**) using scanning electron microscopy (SEM) (**a**,**b**), particle size (**c**,**d**), and FT-IR (**e**,**f**) analysis.

The liquid phase containing *L. sativum* extract’s active principles was efficiently encapsulated in the lipid phase containing hyaluronic acid, phosphatidylcholine, and chia seed fatty acid combinations. The presence of *L. sativum* extract’s active principles was confirmed by GC-MS analysis (Figure S1). The identified major phytochemicals, such as 3-Isoquinolinamine, 5-(hydroxymethyl)-2-Furancarboxaldehyde, 2,3,5,6-Tetrafluoroanisole, 9,12,15-Octadecatrien-1-ol, and (Z)-9-Octadecenamide are presented in Table S1. In Figure 2, the SEM analysis confirmed the particle dispersion size of free LS extract in Figure 2a, and LS extract-loaded solid lipid nanoparticles (LS-SLNp) were confirmed with individual particle morphology with a uniform range between 40 and 80 nm (Figure 2b). Figure 2c shows the availability of a scattered range of particle distribution in Zetasizer for LS extract alone, found between the ranges of 50 to 100 nm, 101 to 1000 nm, and 1001 to 4000 nm. Most interestingly, Figure 2d shows the narrow particle distribution between 9 nm and 230 nm; the average particle size was found in Zetasizer as 72.5 nm for LS-SLNp. In addition, the size distribution of freshly prepared LS-SLNp particles ranges between 9 nm and 230 nm, with the average particle size of 72.5 nm (r.nm) (Figure S2a). The size distribution of freshly prepared LS-SLNp combined with growth medium (0 h), the particle sizes range between 110 nm and 800 nm, with an average particle size of 72.6 nm (r.nm) in Zetasizer (Figure S2b). No changes in the average particle size of fresh and growth media-added LS-SLNp confirmed that there was no aggregation of particles during the time of exposure to cell(s).

The comparison of FT-IR data between LS extract (Figure 2e) and LS-SLNp (Figure 2f) confirmed that there were no significant losses or missing peaks, which confirmed that all the LS extract containing phytochemicals were encapsulated into the LS-SLNp. The functional group’s internalization and variations were confirmed in FT-IR data, with the occurrence of a peak corresponding to the aliphatic primary amine group, such as 3321.2 cm^−1^ (stretching of N-H), 2923.8 cm^−1^ (C-H stretching), 2832.9 (stretching C-H or N-H of amine group), mild shifting of peaks from 2214 to 1999.2 (C=C=O, ketene; N=C=N, carbodiimide; N=C=S, isothiocyanate groups), new peaks from 1602.4, 1541.4, and 1453.6 (C=C stretching of conjugated alkene group, N-H bending of an amine group, C-H bending in alkane of methyl group), and from 1229.6 to 603.9 (C=O stretching of aromatic ester or aldehyde or amine group) in LS-SLNp, confirmed the addition of LS phytochemicals with SLNp as a functional group.

### 4.2. Effect LS and LS-SLNp on hMSCs and SH-SY5Y Cell Proliferation

**Figure 3:**
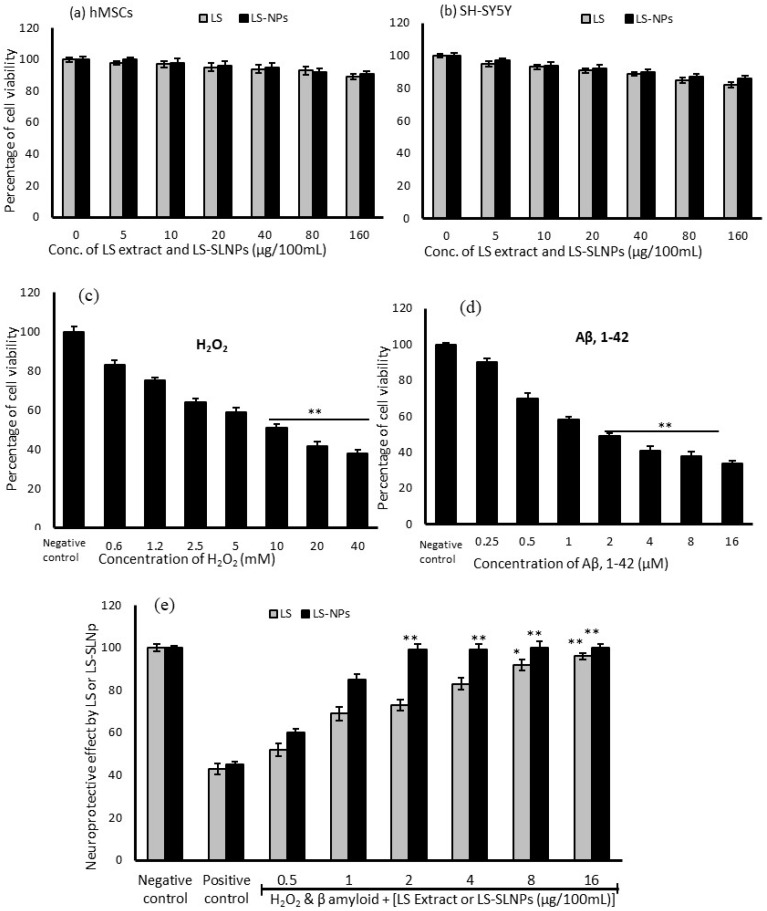
Effect of *L. sativum* extract (LS) and *L. sativum* extract-loaded solid lipid nanoparticles (LS-SLNps) on hMSCs (**a**) and SH-SY5Y cell (**b**) proliferation. The cytotoxic effect of H_2_O_2_ (**c**), beta amyloid [A*β*,1-42] (**d**) after 24 h on SH-SY5Y neuroblastoma cells. The neuroprotective effect of LS and LS-SLNp on H_2_O_2_ and beta amyloid-induced SH-SY5Y neuroblastoma cells (**e**) after 24 h. The values are presented as mean ± SD (n = 6). (**c**,**d**) ** *p ≤* 0.01, by compared with negative control. (**e**) ** *p ≤* 0.01, * *p ≤* 0.05 by compared with positive control.

Freshly prepared LS and LS-SLNp have been used to analyze the biosafety and cytotoxic effect in hMSCs and SH-SY5Y cells. In Figure 3a, the cytotoxicity assay confirmed that even at the highest tested concentration of 160 µg/100 mL of LS alone found a non-significant growth decline in hMSCs. Figure 3b shows the results of cytotoxic effect in SH-SY5Y cells, the LS extract alone with the highest concentration at 160 µg/100 mL was found causing a 14% decline in total cell population. Most notably, LS-SLNp did not demonstrate a significant decline in growth decline until 48 h.

### 4.3. Cytotoxic Effect of H_2_O_2_ and β-Amyloid on SH-SY5Y Neuroblastoma Cells

H_2_O_2_ alone or H_2_O_2_ (10 mM)-induced oxidative-stressed SH-SY5Y cells were incubated with *β*-amyloid [A*β*,1-42] fibrils for 24 h and significantly decreased the cell population, showed changes in the cell morphology, and a gradual decline in mitochondrial oxidative capacity. We found that 40 mM of H_2_O_2_ significantly decreased the SH-SY5Y cell proliferation up to 62% (Figure 3c). Also, 16 µM of A*β*,1-42 significantly reduced the neuronal cell population up to 66% (Figure 3d), confirming the neurotoxic effect. We found the IC_50_ ranges for H_2_O_2_ at 10 mM and A*β*,1-42 at 2 µM concentrations.

### 4.4. Preventative Effect of LS-SLNp against H_2_O_2_ and Aβ,1-42 Induced Toxicity in SH-SY5Y Cells

In SH-SY5Y, the LS-SLNp treatment found with significantly increased cell proliferation with all the tested doses (0.5, 1, 2, 4, 8, and 16 µg/100 mL) was confirmed by the high content of purple formazan crystal (Figure 3e). LS-SLNp pretreatment for 24 h to SH-SY5Y cells exposed to neurotoxic agents such as 10 mM of H_2_O_2_ and 2 µM of *β*-amyloid [A*β*,1-42] fibrils for 24 h effectively prevented the neurotoxicity and found an increased cell proliferation with an increased drug concentration (Figure 3e). The results confirmed that within a 2 µg/100 mL dose of LS-SLNp increased the cell viability to 100%; however, in LS treatment, we found 98% viability in the tested higher dose of 16 µg/100 mL only. The neuronal cell protection potential of LS-SLNp was significantly higher (85%) than LS-alone-(63%)-treated SH-SY5Y cells. We chose 4 µg/100 mL doses for further molecular level studies, which are safe as a human starting dose. The starting dose or human equivalent dose (HED) of nanoparticles (drugs) to humans are calculated based on the maximum recommended starting dose (MRSD), which is given in milligrams per kilogram body weight (mg/kg). This calculation is consistently applied and can be individually determined for each person [21,22,23].

### 4.5. Determination of Neuroprotective Potential via Cell and Nuclear Staining

**Figure 4:**
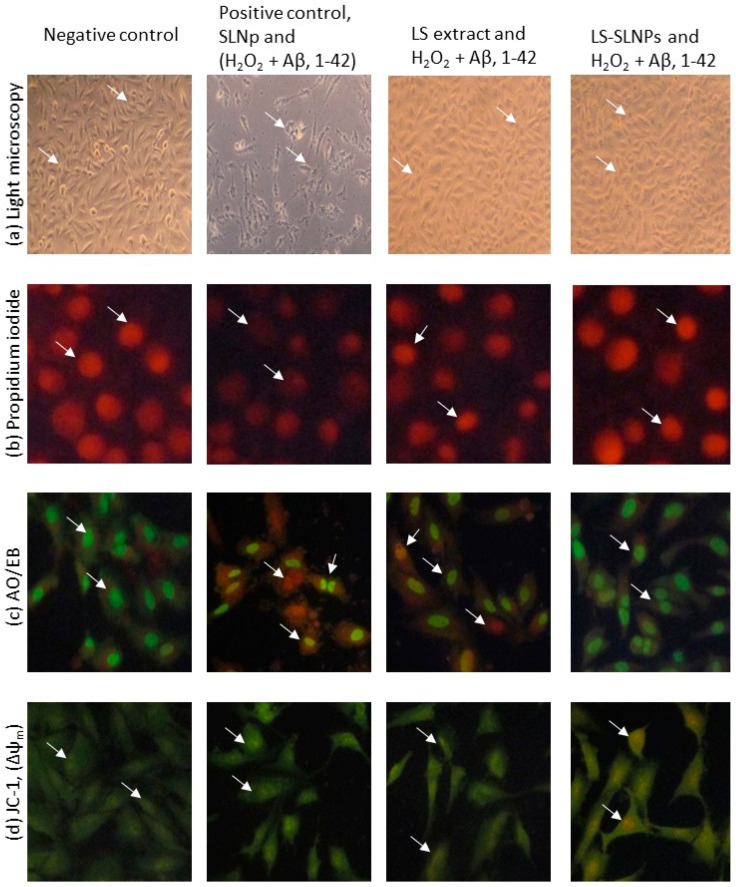
The light microscopic (**a**), florescence microscopic image (200×) analysis of propidium iodide (**b**), AO/EB (**c**), and JC-1 staining (**d**) for negative control, SLNp with H_2_O_2_ and A*β*,1-42-treated positive control, LS extract, or LS-SLNp pretreated (24 h) SH-SY5Y neuroblastoma cells exposed to H_2_O_2_ and A*β*,1-42 for 24 h. PI staining found that the nucleus appeared with signs of pyknosis morphology (signs of apoptosis) in positive control cells (white arrow head) compared to negative control (vehicle-treated). However, cells pretreated with LS-SLNp showed normal morphology with no signs of apoptotic nucleus compared to LS alone. AO/EB staining: positive control cells showing late apoptotic (red), early apoptotic (orange), and pro-apoptotic (bright green) cells (white arrow head); but LS-SLNP-pretreated cells found with dark green (normal cells) color alone. In the JC-1 fluorescence images, merged representations of the red and green signals of the dye indicate the presence of J-aggregates and monomeric forms of JC-1, respectively. Notably, in positive control cells, fewer J-aggregates (indicated by the white arrowhead) were observed compared to the vehicle control group. However, in LS-SLNP-pretreated SH-SY5Y cells, a higher abundance of J-aggregates was evident, directly signifying an elevated mitochondrial membrane potential (high MMP, Δψm) and indicating active mitochondria when compared to cells treated with LS alone.

SH-SY5Y cells exposed to H_2_O_2_ and A*β* (1-42) (positive control), found with an irregular shape of the plasma membrane, uplifted and aggregated cells’ morphology in light microscopic observation are presented with the white arrow head in Figure 4a. However, pretreatment with 4 µg/100 mL doses of LS or LS-SLNp found with both normal and 95% of cells appeared to possess good adherence and a uniform morphology of cell size compared to positive control cells.

Propidium iodide staining found that 85% of oxidatively stressed SH-SY5Y cells treated with A*β* (1-42) were in the irregular shape of the nuclear membrane, and the irregular shape of nuclear content (Figure 4b, white arrow head) confirmed the apoptotic features. The nuclear damage was reversed or normalized in LS or LS-SLNp pretreatment; the percentage of apoptotic cells in LS-SLNp-treated cells was 3% compared to the untreated control (70%).

AO/EB staining of untreated SH-SY5Y cells identified 85% late apoptotic and 10% necrotic cells (Figure 4c, white arrow head). Nevertheless, a 4 µg/100 mL dose of LS-SLNp-pretreated cells was found with 80% normal, 10% early apoptotic, and 5% late apoptotic cells. This 15% of apoptotic cells (early and late) may be due to the H_2_O_2_ and A*β* (1-42)-induced neurotoxicity, which might not reverse the severity completely. LS-SLNp (4 µg/100 mL) produced a significant reversal of apoptosis morphology. The morphology of SH-SY5Y cells pretreated with LS or LS-SLNp was found to be similar to the vehicle-alone-treated negative control cells. The neuroprotective effect of LS-SLNp was significantly higher when compared to LS alone.

### 4.6. Mitochondrial Membrane Potential (Δψm, JC-1)

Typically, healthy cells exhibit a high MMP (Δψm), and this parameter is directly correlated with their oxidative capacity and energy metabolism level. The results obtained with JC-1 dye serve as an indicator of MMP. This is affirmed by the uptake of the cationic, natural green florescent JC-1 dye by the electronegative mitochondria. Subsequently, the dye undergoes internal conversion into irreversible red florescent j-aggregates, further confirming the status of the mitochondrial membrane potential. In the present study, oxidatively stressed SH-SY5Y cells treated with A*β* (1-42) were identified with low mitochondrial membrane polarization, which was confirmed by the lower irreversible J-aggregates or high bright green fluorescence accumulation evidenced by the loss of intra- and extra-mitochondrial ion exchange. LS-SLNp-pretreated SH-SY5Y cells were found with high red fluorescence compared to the untreated control (Figure 4d). The appearance of the red color corresponds to a high accumulation of J-aggregates (white arrow head), evidenced by the high mitochondrial membrane polarity transport of the red-colored JC-1 dye from the outer mitochondrial to inner intramitochondrial J-aggregates.

### 4.7. Changes in Pro-Oxidant and Antioxidant Activity

Table 2 shows the changes in oxidant levels in LS or LS-SLNp-pretreated hMSCs and SH-SY5Y. The results show a significantly (*p* ≤ 0.001) high level of LPO and lower levels of GR, CAT, SOD, and GPX activity in oxidative stress-induced hMSCs and SH-SY5Y (positive control). Pretreated hMSCs and SH-SY5Y cells significantly (*p* ≤ 0.001) reduced the level of LPO, increased the activities of GR, CAT, SOD, and GPX intracellularly. The antioxidant level was found to be higher in LS-SLNp when compared to LS-alone-treated both hMSCs or SH-SY5Y cells.

### 4.8. Alterations in Gene Expression Levels in hMSCs Pretreated with LS-SLNp

**Figure 5:**
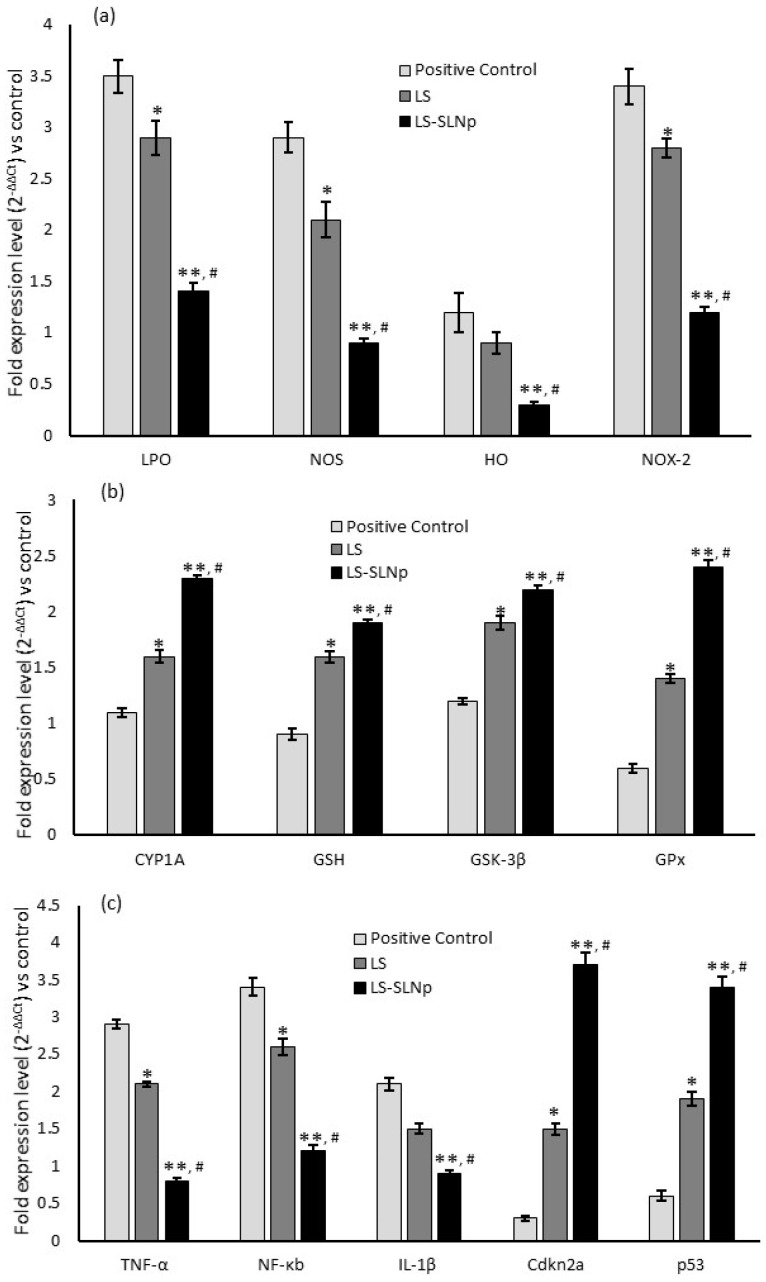
Results show that the alterations in the expression of pro-oxidant (**a**), antioxidant (**b**), pro-inflammatory, and anti-oncogene-associated genes (**c**) in positive control, LS, and LS-SLNp-pretreated (24 h) human mesenchymal stem cells (hMSCs) exposed to H_2_O_2_ for 24 h. The values are presented as mean ± SD (n = 6). * *p ≤* 0.05 and ** *p ≤* 0.001 by comparison with positive control. ^#^ *p ≤* 0.05, comparison of LS-SLNp-pretreated cells with LS-pretreated cells exposed to H_2_O_2_ and A*β*,1-42.

Alterations in mRNA expression of oxidative stress, immunomodulatory, and tumor suppression-related genes were detected in both normal and oxidative-stressed hMSCs following treatment with LS-SLNp. The upregulation in antioxidant and anti-inflammatory gene expressions in LS-SLNp treatment was observed with a significant (*p* ≤ 0.001) downregulation in oxidative stress markers, including LPO, NOS, HO, and NOX-2, in comparison to untreated or H_2_O_2_-alone-treated hMSCs (Figure 5a). Additionally, the expression of CYP1A significantly (*p* ≤ 0.001) increased with LS-SLNp treatment. The stimulation of antioxidant effectiveness of LS-SLNp was further confirmed by elevated expressions of GSH, GSK-3β, and GPx compared to the vehicle control or hMSCs induced with oxidative stress (Figure 5b).

A notable increase in the expression levels of TNF-α and NF-κb was significant in hMSCs subjected to oxidative stress. Furthermore, the expression of IL-1β was significantly increased in both untreated and oxidatively stressed cells, and attained a considerable fold change (Figure 5c). In addition, a two-fold decrease in cdkn2a and p53 expressions associated with tumor suppressor genes compared to oxidatively stressed hMSCs. Notably, the expression levels of pro-inflammatory genes, cellular metabolic inflammatory markers, and tumor suppressor-related genes were restored significantly (*p* ≤ 0.001) in LS-SLNp-pretreated hMSCs.

### 4.9. Neuroprotection-Related Gene Expression Levels in SH-SY5Y Cells Pretreated with LS-SLNp

**Figure 6:**
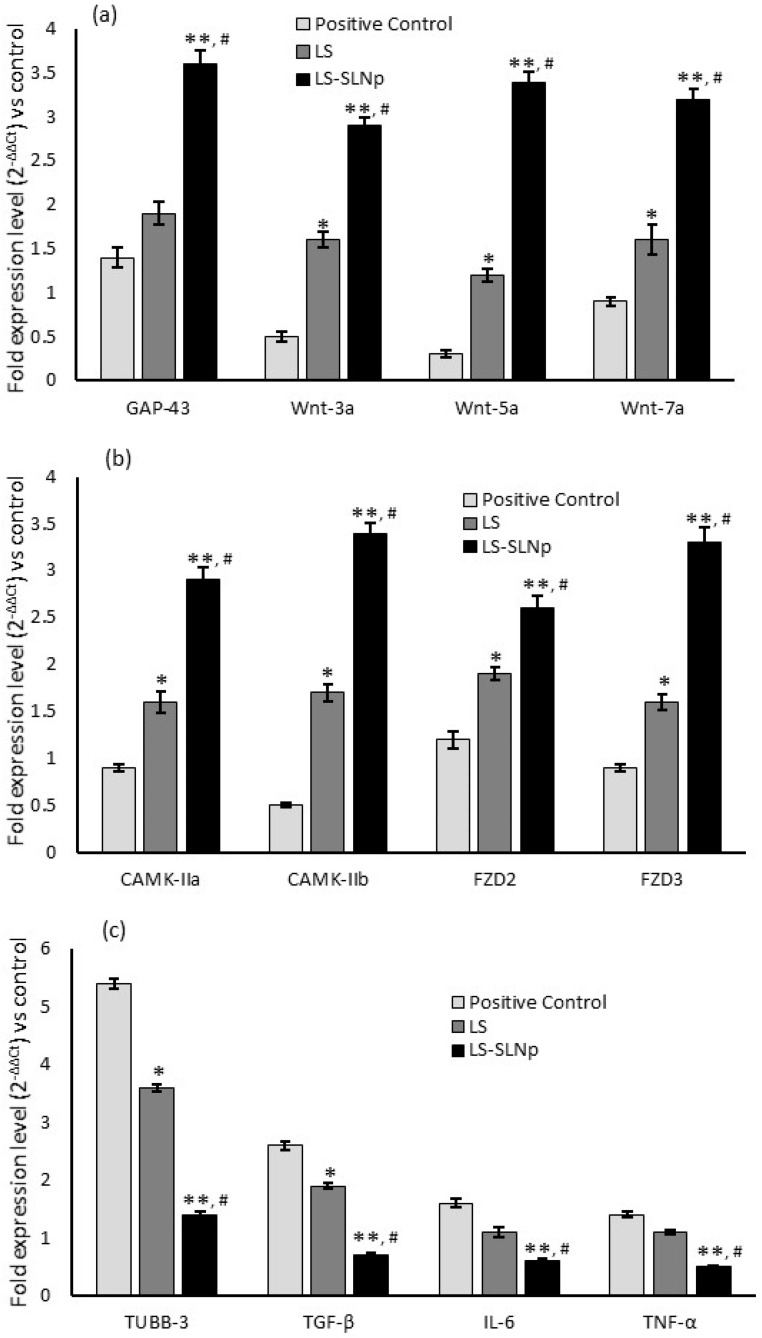
Results showing the alterations in neuronal cell growth-associated (**a**–**c**) gene expression levels in H_2_O_2_ and A*β*,1-42 treated positive control, LS or LS-SLNp-pretreated (24 h) SH-SY5Y neuroblastoma cells exposed to H_2_O_2_ and A*β*,1-42 for 24 h. The values are presented as mean ± SD (n = 6). * *p ≤* 0.05 and ** *p ≤* 0.001 in comparison with positive control. ^#^ *p ≤* 0.05, comparison of LS-SLNp-pretreated cells with LS-pretreated cells exposed to H_2_O_2_ and A*β*,1-42.

Aging and A*β* (1-42)-induced neuronal synapse vulnerability-related mRNA expression levels were quantified in oxidative stress-induced and LS-SLNp-pretreated SH-SY5Y neuroblastoma cells. Neuroblastoma cells (SH-SY5Y) subjected to oxidative stress and treated with A*β* (1-42) for 24 h exhibited a significant reduction in both cell proliferation and population, which was evidenced with the decreased growth associated protein-43 (GAP-43), canonical (Gsk-3*β*)/non-canonical (CAMK-IIa) wingless-related integration site (Wnt) signaling pathway. In addition, we observed an increased expression level of tubulin-associated protein (T-tau) and beta-tubulin protein-3 (TUBB3) receptor genes (Figure 6). It is noteworthy that LS-NPs pretreated SH-SY5Y cells induced with oxidative stress and A*β* (1-42) exposure significantly upregulated the levels of GAP-43, Wnt3a, Wnt5a, Wnt7a, calcium/calmodulin-dependent protein kinase II-a (CAMK-IIa and CAMK-IIb), and Frizzle receptor (FZD2, FZD3) expression (Figure 6a,b). Most notably, LS-SLNp-pretreated SH-SY5Y cells had significantly decreased levels of beta-tubulin protein-3 (TUBB3) (Figure 6c).

### 4.10. Protein Levels

**Figure 7:**
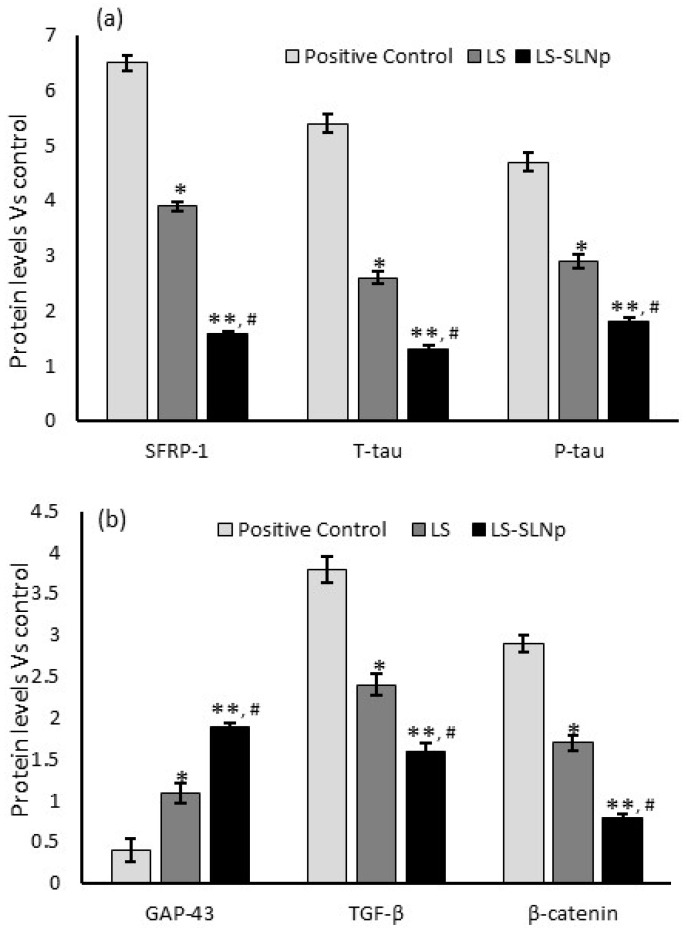
Results showing the alterations in neuronal cell growth-associated (**a**,**b**) protein levels in H_2_O_2_ and A*β*,1-42-treated positive control, LS, or LS-SLNp-pretreated (24 h) SH-SY5Y neuroblastoma cells exposed to H_2_O_2_ and A*β*,1-42 for 24 h. The values are presented as mean ± SD (n = 6). * *p ≤* 0.05 and ** *p ≤* 0.001 in comparison with positive control. ^#^ *p ≤* 0.05, comparison of LS-SLNp pretreated cells with LS pretreated cells exposed to H_2_O_2_ and A*β*,1-42.

The intracellular protein extraction and analysis in LS-SLNPs pretreated SH-SY5Y neuroblastoma cells undergo oxidative stress and A*β* (1-42) exposure. A*β* (1-42) induced synaptic vulnerable neuronal cell’s protein such as secreted frizzled-related protein-1 (SFRP-1), T-tau, P-tau, β-catenin, TGF-β were increased two-fold in untreated SH-SY5Y neuroblastoma cells (Figure 7a,b). However, 24 h of LS-SLNp-pretreated SH-SY5Y cells were found to significantly decrease synaptic vulnerable neuronal cell protein levels and pro-inflammatory TGF-β protein.

## 5. Discussion

Under physiological conditions, neural stem cells (NSCs) differentiate into neuroblasts, then neurons, oligodendrocytes, and astrocytes [24]. Neuroblasts play a significant role during embryonic development. However, they persist into adulthood, contributing to the generation of fresh brain cells and assisting in the recovery process following brain injury and neuronal death [25]. The neuronal cell death is accompanied by an increased unesterified cholesterol release and conversion of cholesterol into the polar metabolite; the present analysis concentrated on evaluating the defenses against oxidative stress, oxysterols, and unesterified cholesterol during the early stages of neuroblast proliferation [26,27]. In this study, our focus was on examining the protective impact of nanoparticles on undifferentiated SH-SY5Y neuroblast cells, specifically before the onset of neuronal cell differentiation.

In addition, understanding the selective neuronal vulnerability (SNV) to oxidative stress is crucial for developing future interventional strategies aimed to protect such susceptible neurons from the challenges associated with the aging process and the pathological conditions leading to neuronal cell degeneration [28]. Currently, many drugs are developed or in progress of development for controlling neurodegenerative diseases; some have failed in clinical trials. The bioavailability of drugs and their ability to traverse the blood–brain barrier (BBB) are pivotal factors in the therapeutic management of AD [29]. In the present study, the edible LS plant metabolites (3-Isoquinolinamine) were fabricated as a solid lipid nanoparticle with hyaluronic acid; the hypothesis has been tested to determine whether they possess bioaccessibility and bioavailability. Hyaluronic acid (HA) is a hydrophilic and biodegradable polymer utilized in LS-SLNPs fabrication. The distinctive characteristics of high molecular weight HA are marked by elevated specificity and biocompatibility, a drug delivery system designed and optimized using HA-SLNs, and the hydrophilic nature of HA meaning it is easily digested by cells which reduces immune rejection or inflammation [30,31].

The preliminary bioefficacy assay confirmed that the fabricated LS-SLNPs support hMSCs cell proliferation and increases in SH-SY5Y neuronal cell growth compared to free LS extract treatment. The dietary polyphenols with fewer side effects pose potential neuroprotective and beneficial effects both in cell and animal models of neurological disorders [11]. In the present study, the identified bioactive metabolites from LS extract, such as 3-Isoquinolinamine [32], triterpene esters, and polyphenols have been identified with neuronal cell protection from *β*-amyloid-induced apoptosis [33]. The components like 2,3,5,6-tetrafluoroanisole and 9-octadecenamide have been found to be beneficial in hippocampal progenitor cell proliferation [34].

In regenerative medicine, the multipotent differentiation potential of hMSCs has been explored as a novel treatment for AD. This is attributed to their ability to target multiple pathological mechanisms associated with AD. In hMSCs, the fabricated LS-SLNPs increased cell proliferation via reduced oxidative stress. The H_2_O_2_-induced oxidative stress was effectively quenched by LS-SLNp and have been confirmed by an increased antioxidant enzyme (SOD, GPX, CAT and GR) activity. The aforementioned findings are substantiated by the elevated expression of antioxidant and pro-apoptotic genes, indicating a mitigation of oxidative stress and restoration of the multilineage capacity of hMSCs at the graft site. The current results affirm a decrease in lipid peroxidative levels (HO, LPO, and NOS) and an increase in antioxidant gene expression (GSH, GSK-3β, and GPX) when compared to the LS extract. This observation confirmed that the fabricated LS-SLNPs possess the highest bioavailability and intracellular uptake mediated by the encapsulated surface lipid. The phytocomponents, 3-isoquinolinamine from LS extract, can be dispersed homogeneously in a HA-containing aqueous phase and encapsulated with phosphatidylcholine and chia seed phospholipids. In this context, HA-SLNPs play a crucial role via enhancing cellular uptake and notably facilitating targeted delivery to specific tumor cancer cells, particularly in expressing CD44^+^ [35]. Hydroxyapatite nanoparticles (HAp-NPs) were fabricated for an effective treatment for Alzheimer’s disease. In silico methods were employed to confirm the biological targets associated with the bioavailability and bioactivity ligands (five), specifically in acetylcholinesterase and butyrylcholinesterase activities [36].

The increase in SH-SY5Y neuronal cell proliferation has been evidenced by the typical morphology of nuclear chromatin content in propidium iodide staining; reduced numbers of pro- and late apoptotic cells in AO/EB fluorescent staining. In addition, the JC-1 assay confirmed the increased mitochondrial membrane potential (Δψm) in LS-SLNP-pretreated SH-SY5Y cells exposed to oxidative stress and A*β*,1-42 amyloid fibrils compared to LS-alone treatment. In this context, Zhang et al. [37] confirmed the protective efficacy of aspirin eugenol ester against paraquat-induced toxicity in SH-SY5Y cells. Zhang et al. [32] have confirmed that 3-Isoquinoline and its derivatives protect neuronal cells from beta-amyloid-induced apoptosis.

The Wnt signaling pathway plays a comprehensive role in neuronal connectivity and synapse formation through the central nervous system (CNS) development, spanning from early stages to adulthood. Wnt is consistently released in the brain to uphold neural activity; and any deregulation in Wnt signaling leads to the onset of neurological disorders [38]. Wnt signaling encompasses a multiplex cascade, classified into canonical or non-canonical pathways [39]. The beta-catenin-dependent canonical pathway, which stimulates long-term synaptic plasticity and cell survival, is regulated by Wnt3a, Wnt7a; and the non-canonical β-catenin independent or calmodulin-dependent protein kinase II (Camk-II) to stimulate postsynaptic compartment is regulated by Wnt4a and Wnt5a [40]. The present study observed that LS-SLNP treatment upregulated the levels of Wnt3a, Wnt7a, and Wnt5a expression, confirming that suppressing A*β*(1-42) fibrils induced neuronal toxicity by canonical or non-canonical pathways. Further, the non-canonical/Wnt signaling cascade Camk-II has been expressed higher, followed by the increased expression of frizzled receptors (FZD2 and FZD3). Previously, Folke et al. [41] observed that the expression of Wnt ligands, specifically Wnt3a, 5a, 7a, and frizzled receptors FZD2 and FZD3, was decreased in the brain of elderly people.

Furthermore, Wnt signaling is implicated in promoting the expression of repressor element-silencing transcription factor-1 (REST) during aging process. In this context, REST represses pro-apoptotic genes, playing a protective role against oxidative stress and the neurotoxic agent, A*β* [42]. Consequently, a downregulated Wnt signaling may contribute to the observed reduction in REST levels in AD [42], and potentially to an increased susceptibility to neurotoxic agent, A*β*. In the human brain with AD, the synaptogenesis role of abnormal aggregation of soluble *β*-amyloid, p-Tau, glia-mediated neuroinflammation, and decreased GAP-43 is well evidenced [43]. Presynaptic dysfunction is characterized by elevated CSF GAP-43 levels in asymptomatic and symptomatic AD patients [44].

Cell proliferation and differentiation have been prominently stimulated by the upregulation of the Wnt/*β*-catenin pathway [45]. In the present study, we found a higher amount of secreted frizzle-related protein 1 (SFRP-1), p-Tau, t-Tau, *β*-catenin, and increased GAP-43 in A*β*,1-42 treated oxidative-stressed SH-SY5Y cells. However, LS-SLNP-treated SH-SY5Y cells stimulated with oxidative stress followed by amyloid fibril treatment found decreased SFRP-1, p-Tau, t-Tau, *β*-catenin levels, and increased GAP-43 protein expression levels. In addition, LS-SLNp effectively quenched the pro-oxidants and increased the antioxidant enzymatic activity in SH-SY5Y cells exposed to oxidative stress. Nalci et al. [46] found that green synthesized magnesium nanoparticles MgS-NPs upregulated antioxidant activity in SH-SY5Y neuroblastoma cells. Overall, the expression of canonical or non-canonical pathway-associated protein and gene expressions were significantly increased by the treatment of LS-SLNp in SH-SY5Y neuroblastoma cells undergoing oxidative stress and followed by the exposure of A*β*,1-42 amyloid fibrils.

## 6. Conclusions

The present study attempted to provide evidence of a substance to protect the neuronal cell from oxidative insult and physiological relevance related to the use of *Lepidium sativum* L., a commonly used medicinal plant. The fabrication of solid lipid nanoparticles improved the absorption, stability, and bioavailability. The findings supported the proliferation of both hMSCs and SH-SY5Y cells. This is accompanied by indications of biosafety, providing a promising avenue for the translation of bioactive components from experimental research in laboratories to potential clinical applications. Our findings suggest that pretreatment with LS-SLNPs to SH-SY5Y cells stimulates Wnt signaling pathway activation and prevents neuronal cell toxicity upon oxidative stress and A*β*,1-42 amyloid fibril exposure. The observed LS-SLNPs effect was more significant than the free LS extract. *Lepidium sativum* L. can be used as a preventive measure for controlling neuronal cell oxidative insults via the Wnt/*β*-catenin/Camk-II signaling mechanism.

## Figures and Tables

**Table 1:** Primer sequences used in quantitative real-time polymerase chain reaction (RT-PCR).

Primer	Forward Sequence (5′ to 3′)	Reverse Sequence (5′ to 3′)
Oxidative stress		
LPO	CTGCCCTATGACAGCAAGAAGC	CGGTTATGCTCGCGGAGAAAGA
NOS	GCTCTACACCTCCAATGTGACC	CTGCCGAGATTTGAGCCTCATG
HO	TCACCTTCCCGAGCATCGAC	TCACCCTGTGCTTGACCTCG
NOX-2	GGAGTTTCAAGATGCGTGGAAACTA	GCCAGACTCAGAGTTGGAGATGCT
GSH	CTGCCGAGATTTGAGCCTCATG	CGGAGTCGAGACAGGTACATGT
GPX	GTGCTCGGCTTCCCGTGCAAC	CTCGAAGAGCATGAAGTTGGGC
GSK-3*β*	GGAACTCCAACAAGGGAGCA	TTCGGGGTCGGAAGACCTT
CYP1A	GCTGACTTCATCCCTATTCTTCG	TTTTGTAGTGCTCCTTGACCATCT
Pro-inflammatory genes		
IL1*β*	CCACAGACCTTCCAGGAGAATG	GTGCAGTTCAGTGATCGTACAGG
IL4	CCGTAACAGACATCTTTGCTGCC	GAGTGTCCTTCTCATGGTGGCT
NF-Kb	GCGCTTCTCTGCCTTCCTTA	TCTTCAGGTTTGATGCCCCC
TNF-α	CTCTTCTGCCTGCTGCACTTTG	ATGGGCTACAGGCTTGTCACTC
p53	CCTCAGCATCTTATCCGAGTGG	TGGATGGTGGTACAGTCAGAGC
PRb_2_	CTCGTGCTGATGCTACTGAGGA	GGTCGGCGCAGTTGGGCTCC
Cdkn-2A	CCTTCCAATGACTCCCTCC	TCAGAAACCCTAGTTCAAAGGA
Nerve cell proliferation-related gene		
GAP-43	AGGGAGAAGGCACCACTACT	GGAGGACGGCGAGTTATCAG
Wnt-3a	TCTACGACGTGCACACCTG	CCTGCCTTCAGGTAGGAGTT
Wnt-5a	AGCAGACGTTTCGGCTACAG	TGCCCCCAGTTCATTCACAC
Wnt-7a	GCGTCTCGCACACTTGCAC	CCGCGCTTTCCGGTTCATAG
Camk-IIa	CATGGTTTGGGTTTGCAGGG	CCGGCTTTGATGCTGGTA
Camk-IIb	GAGGACGGAGCAGTGTCTAA	GACGCACGATGTTGGAATGC
TUBB3	CCGAAGCCAGCAGTGTCTAA	AGGCCTGGAGCTGCAATAAG
FZD2	TCCATCTGGTGGGTGATTCTG	CTCGTGGCCCCACTTCATT
FZD3	GCCTATAGCGAGTGTTCAAAACTCA	TGGAAACCTACTGCACTCCATATCT
Housekeeping gene		
*β* Actin	GATCTTGATCTTCATGGTGCTAGG	TTGTAACCAACTGGGACCATATGG

**Table 2:** Changes in the cellular LPO level and antioxidant enzyme (GR activity, SOD activity, CAT activity, and GPx activity) in positive control, LS, or LS-SLNp-pretreated hMSCs or SH-SY5Y neuroblastoma cells exposed to oxidative stress. The values are presented as mean ± SD (n = 6). * *p* ≤ 0.05 and ** *p* ≤ 0.001 in comparison with positive control. ^#^ *p* ≤ 0.05 comparison of LS-SLNp pretreated cells with LS-pretreated cells exposed to H_2_O_2_ and A*β*,1-42. [Units: LPO—expressed as *µ* moles of MDA formed/gram; GR activity—one unit defined as the reduction of 1 μmol/min GSSG. SOD activity—one unit corresponds to the quantity of the enzyme in 20 μL of the sample solution that inhibits the reduction reaction of WST-8 with superoxide anion by 50%. CAT activity—the amount of the enzyme that can catalyze 1 μM H_2_O_2_ within 1 min under the condition of pH-7. GPx activity—the activity was expressed as the conversion of 1 mM/min NADPH to NADP+.

	hMSCs			SH-SY5Y Cells		
	Positive Control	LS	LS-SLNp	Positive Control	LS	LS-SLNp
LPO	0.9 ± 0.02	0.6 ± 0.01 *	0.4 ± 0.02 *, ^#^	0.5 ± 0.01	0.3 ± 0.01 *	0.06 ± 0.01 **, ^#^
GR	2.1 ± 0.01	3.2 ± 0.2 **	3.9 ± 0.01 *	0.06 ± 0.03	1.3 ± 0.012 *	2.05 ± 0.02 **, ^#^
SOD	1.5 ± 0.01	2.9 ± 0.15 *	4.2 ± 0.03 **, ^#^	0.03 ± 0.01	0.9 ± 0.01 *	1.5 ± 0.01 **, ^#^
CAT	1.7 ± 0.02	2.3 ± 0.09 *	2.9 ± 0.01 *, ^#^	0.05 ± 0.01	1.3 ± 0.02 *	2.6 ± 0.03 **, ^#^
GPX	1.6 ± 0.01	2.04 ± 0.11 *	3.1 ± 0.012 **, ^#^	1.1 ± 0.02	2.3 ± 0.013 *	2.9 ± 0.025 *, ^#^

## Data Availability

The data presented in this study are available in the Supplementary Materials.

## Supplementary Materials

The following supporting information can be downloaded at: https://www.mdpi.com/article/10.3390/nano14020199/s1. Figure S1: gas chromatography–mass spectrum (GC-MS) chromatogram for *L. sativum* L. extract; Table S1: GC-MS phytochemical profiling of *L. sativum* methanol extract (LSE) show 99–95% similarity in the NIST-11 database; Figure S2: analysis of particle size using size distribution by intensity for *L. sativum* extract-loaded solid lipid nanoparticle (LS-SLNp) with free (Figure S2a) and growth media added (0 h) (Figure S2b) conditions.
