# Supplementary material for: Neuroprotective Effect of Solid Lipid Nanoparticles Loaded with Lepidium sativum (L.) Seed Bioactive Components Enhance Bioavailability and Wnt/β-Catenin/Camk-II Signaling Cascade in SH-SY5Y Neuroblastoma Cells
Source: Nanomaterials (Basel). 2024 Jan 16;14(2):199. doi: 10.3390/nano14020199 (PMC10819627; doi:10.3390/nano14020199)
Supplement: Supplementary file 1 [file nanomaterials-14-00199-s001.zip › nanomaterials-2758483-supplementary.pdf]

Supplementary material

Supplementary Figures

Figure S1. Gas chromatography- mass spectrum (GC-MS) chromatogram for *L. sativum* L. extract.

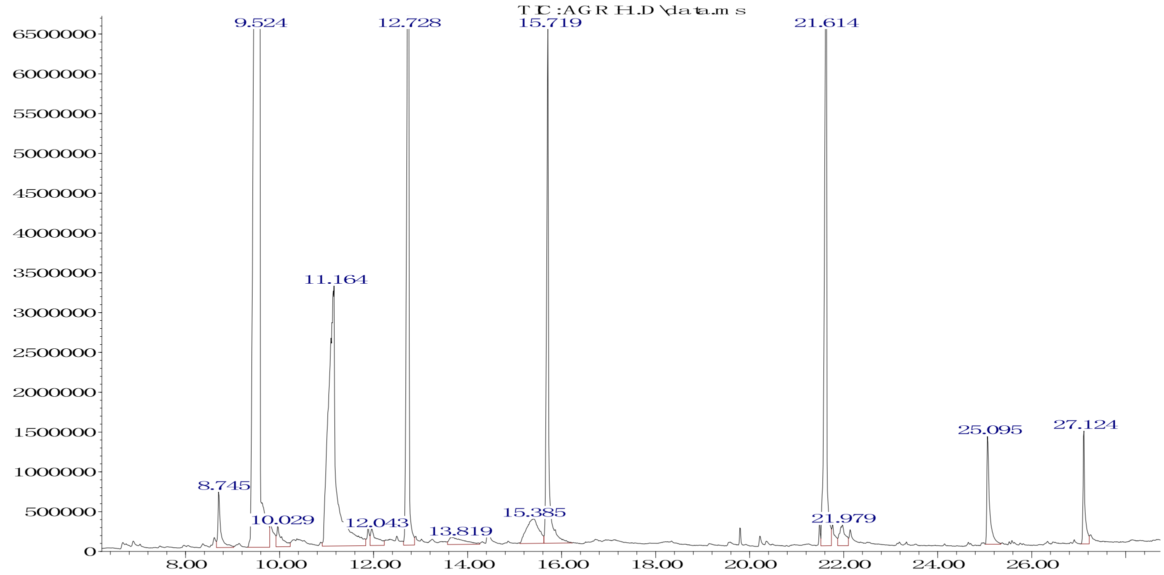

All data were collected after the full-scan mass spectra between 40-550 AMU scan range. The presence of *L. sativum* extract's active principles was confirmed by GC-MS analysis (supplementary figure s1). LabSolution software (Shimadsu, Tokyo, Japan) have be used to control the operation of GCMS. The obtained peak(s) corresponding mass spectra were identified and compared using the National Institute of Standard Technology (NIST-11) library.

Table S1. GC-MS phytochemical profiling of *L. sativum* methanol extract (LSE) shown 99–95% similarity in the NIST-11 database.

S. No	Peak Width 50% (min)	Hit Name	Mol. Weight (Atomic mass unit)
1	0.369	Benzoic acid, methyl ester	136.052
2	0.473	Benzyl nitrile	117.058
3	0.303	3-Isoquinolinamine	144.069
4	0.927	5-(hydroxymethyl)-2-Furancarboxaldehyde,	126.032
5	0.303	6-Aminotetrazolo(b)pyridazine	136.05
6	0.236	(isothiocyanatomethyl)-Benzene,	149.03
7	0.69	Benzeneacetamide	135.068
8	0.511	1,6-anhydro-beta.-D-Glucopyranose	162.053
9	0.605	2,3,5,6-Tetrafluoroanisole	180.02
10	0.227	3-(3,4,5-trimethoxyphenyl)-2-Propenoic acid,	238.084
11	0.227	(Z,Z,Z)-9,12,15-Octadecatrien-1-ol,	264.245
12	0.331	2-dimethylaminoethyl ester 4-Butylbenzoic acid,	249.173
13	0.18	(Z)-9-Octadecenamide,	281.272

The obtained peak(s) in GC-MS and its corresponding mass spectra were identified and compared using the National Institute of Standard Technology (NIST-11) library. The identified major phytochemicals, such as 3-Isoquinolinamine, 5-(hydroxymethyl)-2-Furancarboxaldehyde, 2,3,5,6-Tetrafluoroanisole, 9,12,15-Octadecatrien-1-ol and (Z)-9-Octadecenamide were presented in supplementary Table s1. The percentage composition of LS or SH constituents was expressed as a peak area percentage.

Figure S2. Analysis of particle size using size distribution by intensity for *L. sativum* extract loaded solid lipid nanoparticle (LS-SLNp) with free [Fig. S2 (a)] and growth media added (0 hr) [Fig. S2 (b)] conditions.

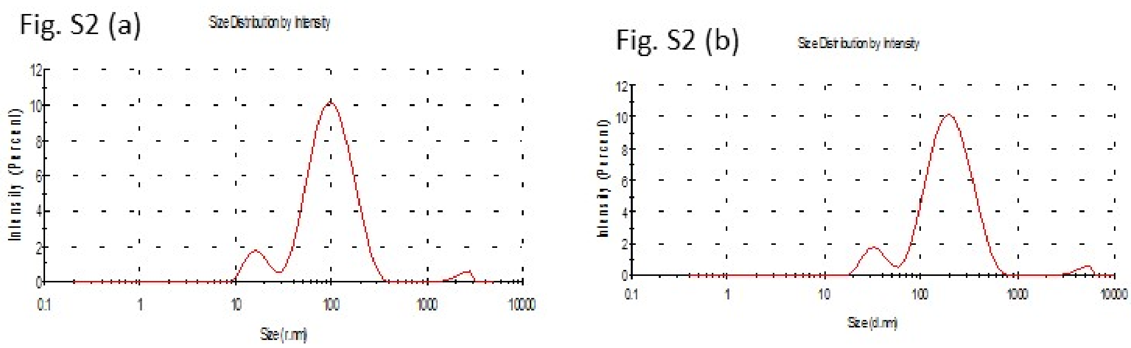

Fig. S2 (a): The size distribution of freshly prepared LS-SLNp particles ranges between 9nm to 230nm, with the average particle size has been found in Zetasizer as 72.5nm (r.nm) for LS-SLNp.

Fig. S2 (b): The size distribution of freshly prepared LS-SLNp combined with growth medium (0hr) particles ranges between 110nm to 800nm, with the average particle size has been found in Zetasizer as 72.6nm (r.nm) for LS-SLNp.

We found with no changes in the particle size of fresh and growth media added LS-SLNp (during 0hr time period of synthesis or treatment).

Methodology: pro and antioxidant assay

Experimental design and test sample preparation

Pretreated hMSCs and SH-SY5Y cells with 4 µg/100mL of LS or LS-SLNp present in 24 well plate, respective cells were exposed to oxidative stress using H_2O_2 for hMSCs, and 10mM of H_2O_2 & 2µM of Aβ,1-42 for SH-SY5Y for 24h. The cells in different treatment condition were labelled accordingly, then the cells were lysed in ice cold lysis buffer (pH 7.4, 0.1M Tris/HCL added with 0.5% Triton X-100, 5mM β-mercaptoethanol, 0.1mg/ml serine protease inhibitor phenylmethylsulfonylfluoride). The cell lysate was collected in labelled microcentrifuge tubes and centrifuged at 14000x g for 5min at 4˚C. The supernatant was used to analyzed the levels of LPO, activities of glutathione reductase (GR), catalase (CAT), superoxide dismutase (SOD) and glutathione peroxidase (GPX) using an enzymatic commercial analytical kit (Sigma-Aldrich, St.louis, MO, USA) using multiwell plate reader.

Measurement of Lipid Peroxidation (LPO).

The malondialdehyde (MDA) content, a measure of LPO, was assayed in the form of thiobarbituric acid-reactive substances (TBARS) according to the sigma kit protocol. In this kit, lipid peroxidation is determined by the reaction of MDA with thiobarbituric acid (TBA) to form a colorimetric (532 nm)/fluorometric (λex = 532/λem = 553 nm) product, proportional to the MDA present. The rate of lipid peroxidation was expressed as μ moles of MDA formed/gram wet weight of tissue.

Assay of Glutathione Reductase (GR) Activity.

Glutathione reductase (GR) activity was determined using the method described by the Sigma glutathione reductase Assay Kit. In brief, 20 µL cell lysate was added to 100 mM PBS (0.85 mL, pH 7.5) containing 1 mM EDTA and 2.32 mM GSSG. NADPH (2 mM, 10 µL) was mixed with the solution and the absorbance of 340 nm was read every 1 min for 5 min at 25°C using a Multi-Detection Microplate Reader (Bio-Tek, Synergy HT, USA). Changes in the rate of absorbance were converted into units of GR per milligram of protein using a molar extinction coefficient of 6.22 mM/cm. One unit of activity was defined as the reduction of 1µmol/min GSSG.

Catalase Assay

In order to measure the level of catalase in cell lysate, the catalase activity assay colorimetric kit was used according to the manufacturer's protocol. Absorbance was measured at 520 nm and results were expressed as units milligram of protein. One unit of catalase is defined as the amount of the enzyme that can catalyze 1 μmol H_2O_2 within 1 minute under the condition of pH-7.

Superoxide Dismutase Assay

SOD activity was investigated utilizing a Total Superoxide Dismutase Assay Kit according to the manufacture's protocol. The water-soluble tetrazolium-8 (WST-8) method, which is more stable and sensitive, is currently used in the determination of SOD activity. Absorbance was determined at 450 nm. Results were expressed as units milligram of protein where protein content was determined by the BCA method. One unit of SOD is defined as the quantity of the enzyme in 20μL of the sample solution that inhibits the reduction reaction of WST-8 with superoxide anion by 50%.

Glutathione Peroxidase (GPx) Activity

GPx was assessed according to the procedure of the cellular glutathione peroxidase assay kit protocol. Briefly, 10 μL cell lysate was mixed with 175μL 100mM potassium phosphate buffer (pH 7.0) containing 1mM EDTA and 11μL of GR solution which contains 30mM -NADPH, 84mM -GSH, and 1μL -5U/μLGR. 4μL of 15mM H_2O_2 was added to the solution mixture. The absorbance was monitored at 340nm every 2 min for 10 min. The activity of GPx within the cell was calculated using a standard curve and expressed as milliunit of GPx per milligram of protein. One unit of activity was expressed as the conversion of 1 mM/min NADPH to NADP+.
